# Atrial fibrillation patterns and their cardiovascular risk profiles in the general population: the Rotterdam study

**DOI:** 10.1007/s00392-022-02071-6

**Published:** 2022-08-10

**Authors:** Martijn J. Tilly, Zuolin Lu, Sven Geurts, M. Arfan Ikram, Bruno H. Stricker, Jan A. Kors, Moniek P. M. de Maat, Natasja M. S. de Groot, Maryam Kavousi

**Affiliations:** 1 Department of Epidemiology, Erasmus MC University Medical Center, Office Na-2714, PO Box 2040, 3000 CA Rotterdam, The Netherlands; 2 Department of Medical Informatics, Erasmus University Medical Center, Rotterdam, The Netherlands; 3 Department of Hematology, Erasmus University Medical Center, Rotterdam, The Netherlands; 4 Department of Cardiology, Erasmus University Medical Center, Rotterdam, The Netherlands

**Keywords:** Atrial fibrillation, Atrial fibrillation patterns, Risk factors, Arrhythmia, Anthropometrics, Repeated measurements

## Abstract

**Background:**

Clinical guidelines categorize atrial fibrillation (AF) based on the temporality of AF events. Due to its dependence on event duration, this classification is not applicable to population-based cohort settings. We aimed to develop a simple and standardized method to classify AF patterns at population level. Additionally, we compared the longitudinal trajectories of cardiovascular risk factors preceding the AF patterns, and between men and women.

**Methods:**

Between 1990 and 2014, participants from the population-based Rotterdam study were followed for AF status, and categorized into ‘single-documented AF episode’, ‘multiple-documented AF episodes’, or ‘long-standing persistent AF’. Using repeated measurements we created linear mixed-effects models to assess the longitudinal evolution of risk factors prior to AF diagnosis.

**Results:**

We included 14,061 participants (59.1% women, mean age 65.4 ± 10.2 years). After a median follow-up of 9.4 years (interquartile range 8.27), 1,137 (8.1%) participants were categorized as ‘single-documented AF episode’, 208 (1.5%) as ‘multiple-documented AF episodes’, and 57 (0.4%) as ‘long-standing persistent AF’. In men, we found poorer trajectories of weight and waist circumference preceding ‘long-standing persistent AF’ as compared to the other patterns. In women, we found worse trajectories of all risk factors between ‘long-standing persistent AF’ and the other patterns.

**Conclusion:**

We developed a standardized method to classify AF patterns in the general population. Participants categorized as ‘long-standing persistent AF’ showed poorer trajectories of cardiovascular risk factors prior to AF diagnosis, as compared to the other patterns. Our findings highlight sex differences in AF pathophysiology and provide insight into possible risk factors of AF patterns.

**Graphical abstract:**

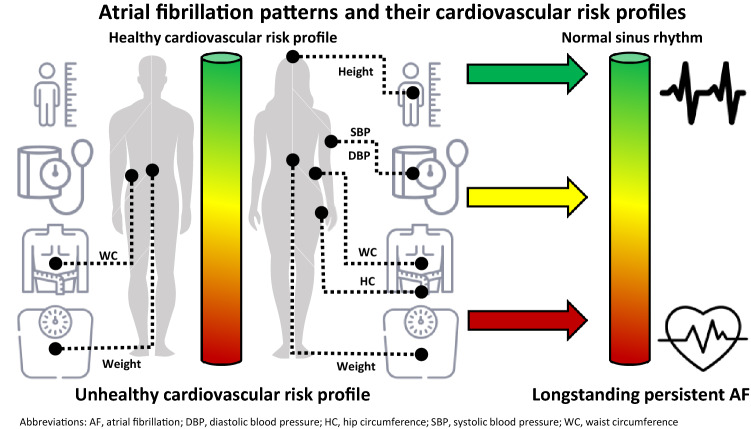

## Background

Atrial fibrillation (AF) is the most common cardiac arrhythmia, with a lifetime risk of almost 25% at the age of 55, and is associated with cardiovascular morbidity and increased mortality [1–4]. With aging of the population, the number of adults with AF is expected to steeply increase in the next decades [5]. AF presentation varies from asymptomatic short AF episodes to persistent AF causing hemodynamic instability [1]. Based on the European Society of Cardiology and American Heart Association guidelines, AF is commonly categorized into five patterns: first diagnosed AF, paroxysmal AF, persistent AF, long-standing-persistent AF, and permanent AF [6, 7]. Clinical studies have provided evidence for differences in the risk of morbidity and mortality between these AF patterns [8–10]. Moreover, cardiovascular risk factors may be associated with AF progression and transition from paroxysmal to persistent AF [11–15].

While this classification is comprehensive in clinical settings, the definitions often vary in research settings [16]. Moreover, due to the large time intervals between study examinations in large longitudinal cohort studies, asymptomatic AF patterns could be missed, and no data are available on the temporality of AF events. The Framingham Heart Study and the PREVEND study developed a classification system for cohort studies based on two-year follow-up [17, 18]. However, the short follow-up could lead to substantial misclassification bias, as later events remain undiagnosed.

Recent literature suggests differences in AF pathophysiology between men and women [19]. Women are often older at the time of diagnosis and have a higher prevalence of hypertension and valvular heart disease. While decisive evidence is lacking, the structural development of AF is suggested to differ, as women often have more atrial fibrosis and distinct patterns in electrical function. This can imply differences in underlying pathophysiology between men and women.

Using data from the Rotterdam study, with a follow-up of up to 24 years, we aimed to develop a simple and standardized method to identify AF patterns in a general population. Additionally, we assessed if the longitudinal trajectories of cardiovascular risk factors preceding AF patterns differ, and evaluated the existence of potential sex differences in AF risk factors.

## Methods

### Study population

The Rotterdam study (RS) is a large ongoing prospective population-based cohort study [20]. In 1990, inhabitants of Ommoord, a suburb in Rotterdam, the Netherlands, aged ≥ 55 years were invited to participate. Out of 10,215 eligible individuals, 7983 were included (RS-I). In 2000, a second cohort consisting of 3011 out of 4504 invitees aged ≥ 55 years was started (RS-II). 3932 out of 6057 individuals aged ≥ 45 years started in the third cohort in 2006 (RS-III). We included all participants from RS-I, RS-II, and RS-III for the classification of AF patterns. Out of 14,926 participants, 306 did not give informed consent for follow-up data collection. Additionally, 559 participants were excluded based on prevalent AF at inclusion. The RS has been approved by the Medical Ethics Committee of the Erasmus MC (registration number MEC 02.1015) and by the Dutch Ministry of Health, Welfare and Sport (Population Screening Act WBO, license number 1071272-159521-PG). The Rotterdam Study Personal Registration data collection is filed with the Erasmus MC Data Protection Officer under registration number EMC1712001. The Rotterdam study has been entered into the Netherlands National Trial Register (NTR; www.trialregister.nl/) and into the WHO International Clinical Trials Registry Platform (ICTRP; www.who.int/ictrp/network/primary/en/) under shared catalog number NTR6831. All participants provided written informed consent to participate in the study and to have their information obtained from treating physicians.

### Assessment of atrial fibrillation

Prevalent AF was assessed at baseline using interviews by trained research assistants and extensive review of the medical records. Ten second 12-lead electrocardiograms (ECGs) were obtained from participants at baseline and during follow-up examinations, stored digitally with an ACTA Gnosis IV ECG recorder (Esaote; Biomedical, Florence Italy) and analyzed using the Modular ECG Analysis System (MEANS) software [21]. All ECG diagnoses were verified by two research physicians blind to the MEANS diagnosis. A cardiologist was consulted when consensus was not reached. To ensure AF events occurring in between the research visits were not missed, besides the periodical research examinations at the research center, the medical databases of general practitioners and hospitals were continuously monitored for reports of (sporadic) AF episodes. Those events occurring in between the research visits were AF events during follow-up were recorded. AF during the process of dying, following myocardial infarction, or following cardiac surgery were not considered events. All participants were followed from inclusion date until January 1, 2014, loss-to-follow-up, or date of death, whichever came first.

### Assessment of risk factors

At baseline and follow-up examinations, participants were measured and weighted without shoes or heavy garments. Body mass index (BMI) was defined as weight in kilograms, divided by the square of height in meters (kg/m^2^). Waist circumference was measured in a standing position during expiration, at the midpoint between the lower rib margin and iliac crest. Hip circumference was measured at the widest point of the hips. We calculated waist-to-hip ratio (WHR) by dividing waist circumference by hip circumference. Systolic (SBP) and diastolic blood pressure (DBP) were defined as the mean of two measurements of the right arm using a sphygmomanometer. Fasting glucose, total cholesterol, and high-density lipoprotein (HDL) cholesterol were measured using standard laboratory techniques. Follow-up for cardiovascular risk trajectories lasted until the first documented AF event, loss-to-follow-up, date of death, or January 1, 2014, whichever came first.

### Atrial fibrillation classification

**Fig. 1:**
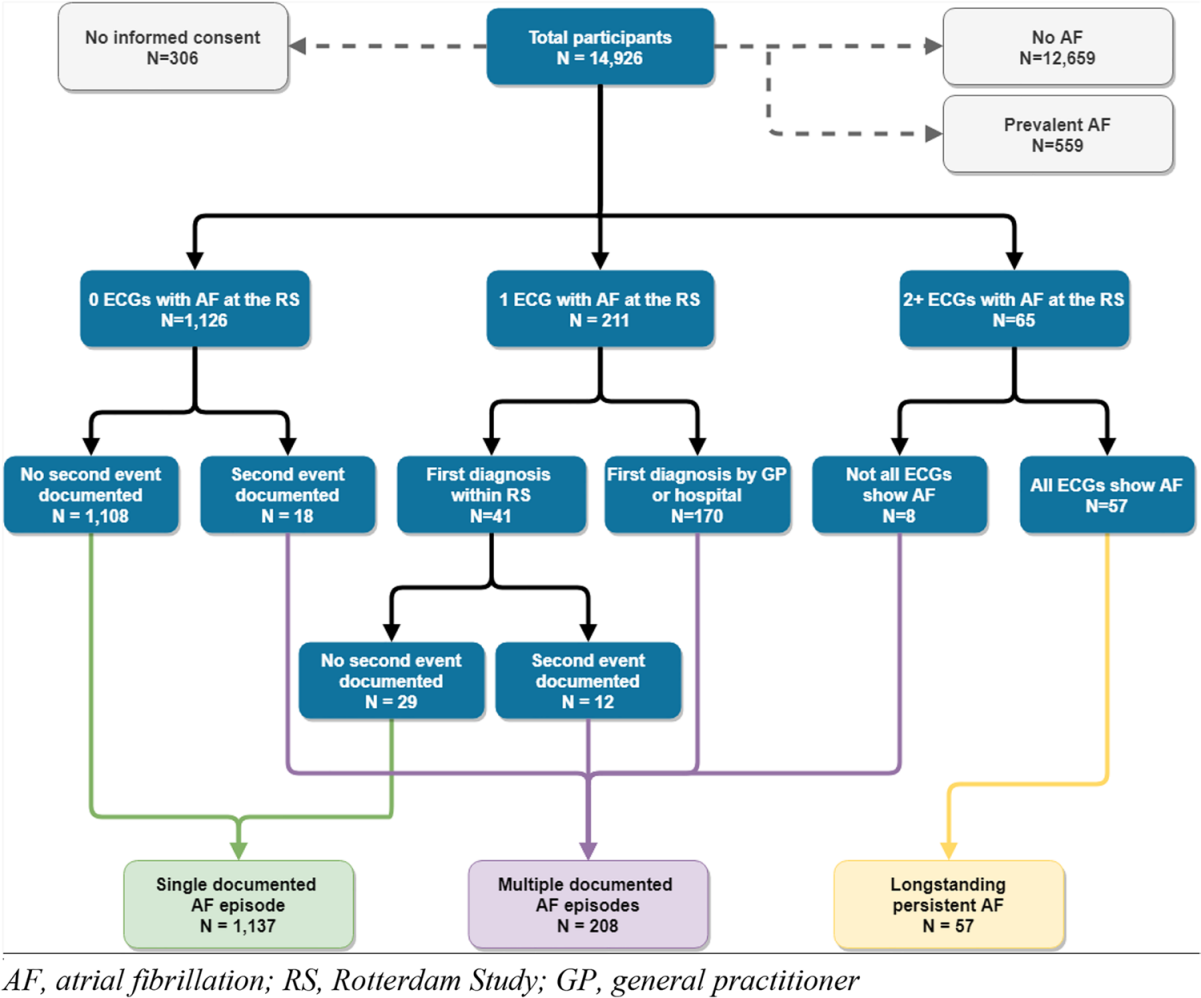
Flowchart of the atrial fibrillation classification of participants in the Rotterdam study. *AF* atrial fibrillation, *RS* Rotterdam study, *GP* general practitioner

Up to six ECGs were available for each participant from the examination rounds. If a single AF episode was reported by the general practitioner, and no ECGs at the examination center showed AF, participants were categorized as ‘single-documented AF episode’. If a second AF event was reported, or at least one additional ECG at the examination center showed AF, participants were categorized as ‘multiple-documented AF episodes’. ‘Long-standing persistent AF’ was defined as at least two consecutive ECGs at the examination center showing AF, not followed by an ECG showing normal rhythm. As on average, the interval between ECGs is five years, it is unlikely that participants in this latter category suffered from two separate events on the exact examination dates. The clinical definition of paroxysmal AF entails an episode duration of ≤ 7 days, therefore, second events were only included if occurring more than seven days after the initial AF event. In total, 1137 participants were categorized as ‘single-documented AF episode’, 208 as ‘multiple-documented AF episodes’, and 57 as ‘long-standing persistent AF’. A graphical overview of the AF classification is provided in Fig. 1.

### Statistical analyses

Baseline characteristics are presented as counts and percentages, mean and standard deviation (SD), or median and interquartile range (IQR), as appropriate. To assess differences at baseline between different AF patterns, one-way ANOVA, independent-sample Kruskal–Wallis, or chi-square tests were performed. Linear mixed-effects models were fitted to assess the longitudinal evolution of each risk factor prior to AF. Age, sex, and cohort were treated as fixed effects in all models, and age was used as timescale. Each model included random intercepts and slopes, and an unstructured covariance matrix. In addition, natural cubic splines with up to three knots for age were added in the models to investigate nonlinearity. Likelihood ratio tests were used to determine the best model for the analyses. Subsequently, the final models were plotted to show the longitudinal trajectories of risk factors among AF patterns, for men and women separately. Complete cases were used for statistical analyses (range of missingness: 0.0–3.4%). Data management and statistical analyses were performed in R, version 4.0.3 (R Foundation for Statistical Computing, Vienna, Austria) and IBM SPSS Statistics for Windows, version 25.0 (IBM Corp., Armonk, New York, USA). A two-tailed *p* value < 0.05 was denoted as statistically significant.

## Results

### Baseline characteristics

We included 14,061 participants (59.1% women, mean age 65.43 ± 10.21 years). As is visible in Table 1, at baseline, women were significantly older (66.17 ± 10.76 vs 64.35 ± 9.26 years) and had a higher BMI (27.14 ± 4.48 vs 26.56 ± 3.52 kg/m^2^) than men. Median total cholesterol (6.37 [5.13–7.61] vs 5.90 [4.72–7.08] mmol/L) and HDL cholesterol (1.49 [1.09–1.89] vs 1.22 [0.89–1.55] mmol/L) levels were also higher in women. Men had significantly higher median SBP (139.01 [118.33–159.69] vs 137.81 [115.61–161.01] mmHg) and DBP (78.59 [66.66–90.52] vs 76.49 [64.65–88.33] mmHg).

**Table 1:** Baseline characteristics of the study population

	Total (*N* = 14,061)	Women (*N* = 8310)	Men (*N* = 5751)	*P* value
Age (years)	65.43 (10.21)	66.17 (10.76)	64.35 (9.26)	< 0.001
Weight (kg)	76.00 (13.82)	71.48 (82.24)	82.24 (12.84)	< 0.001
Height (cm)	167.96 (9.57)	162.26 (6.70)	175.83 (7.03)	< 0.001
BMI (kg/m^2^)	26.90 (4.11)	27.14 (4.48)	26.56 (3.52)	< 0.001
WC (cm)	92.03 (11.96)	88.71 (11.89)	96.61 (10.46)	< 0.001
HC (cm)	102.70 (8.85)	103.51 (9.59)	101.58 (7.57)	< 0.001
WHR	0.90 (0.09)	0.86 (0.08)	0.95 (0.07)	< 0.001
SBP (mmHg)	138.31 (21.58)	137.81 (22.20)	139.01 (20.68)	< 0.01
DBP (mmHg)	77.36 (11.93)	76.49 (11.84)	78.59 (11.93)	< 0.001
Prevalent DM, *N* (%)	1352 (12.7%)	687 (11.2%)	665 (14.8%)	< 0.001
Total chol (mmol/L)	6.17 (1.24)	6.37 (1.24)	5.90 (1.18)	< 0.001
HDL-chol (mmol/L)	1.38 (0.39)	1.49 (0.40)	1.22 (0.33)	< 0.001
Use of cardiac medication, *N* (%)	980 (7.0%)	566 (6.8%)	414 (7.2%)	0.435
Use of lipid-lowering medication, *N* (%)	1353 (9.6%)	704 (8.5%)	649 (11.3%)	< 0.001
Current smoking, *N* (%)	3378 (24.5%)	1663 (20.5%)	1715 (30.2%)	< 0.001

Data as *N* (%) or mean ± SD. *P* value based on *χ*^2^ for categorical data, or independent samples *T*-test for continuous data. *P* values refer to differences in baseline risk factors between women and men

*AF* atrial fibrillation, *BMI* body mass index, *WC* waist circumference, *HC* hip circumference, *WHR* waist-to-hip ratio, *SBP* systolic blood pressure, *DBP* diastolic blood pressure, *DM* diabetes mellitus, *chol* cholesterol, *HDL* high-density lipoprotein

After a median follow-up time of 9.4 (8.3) years, 1402 participants (10.0%) developed a first AF event, out of which 1137 (81.1%) were categorized as ‘single-documented AF episode’, 208 (14.8%) as ‘multiple-documented AF episodes’, and 57 (4.1%) participants as ‘long-standing persistent AF’. In total, 4953 participants died during follow-up, and 406 (2.9%) participants were lost-to-follow-up due to different reasons. The proportion of women decreased toward the more severe AF categories; 54.7% (622 women vs 515 men) in ‘single-documented AF episode’, 49.5% (103 vs 105) in ‘multiple-documented AF episodes’, and 38.6% (22 vs 35) in ‘long-standing persistent AF’. As shown in Table 2, there were significant differences between the AF patterns for all risk factors of interest at baseline, except hip circumference.

**Table 2:** Baseline characteristics of the study population per atrial fibrillation pattern

	No AF (*N* = 12,659)	Single-documented AF episode (*N* = 1137)	Multiple-documented AF episodes (*N* = 208)	Long-standing persistent AF (*N* = 57)	*P* value
Women, *N* (%)	7563 (59.7)	622 (54.7)	103 (49.5)	22 (38.6)	< 0.001
Age (years)	65.10 (10.35)	68.54 (8.32)	67.29 (8.58)	64.94 (6.72)	< 0.001
Weight (kg)	75.80 (13.89)	77.2 (13.00)	78.23 (13.34)	83.23 (12.92)	< 0.001
Height (cm)	167.87 (9.54)	168.43 (9.86)	169.12 (9.77)	172.73 (9.34)	< 0.001
BMI (kg/m^2^)	26.85 (4.13)	27.22 (3.98)	27.32 (3.90)	27.87 (3.96)	< 0.01
WC (cm)	91.84 (11.96)	93.72 (11.88)	92.57 (11.17)	95.52 (13.03)	< 0.001
HC (cm)	102.76 (8.92)	102.28 (8.32)	101.50 (8.26)	103.30 (7.78)	0.08
WHR	0.89 (0.09)	0.92 (0.09)	0.91 (0.09)	0.92 (0.10)	< 0.001
SBP (mmHg)	137.64 (21.36)	143.91 (22.29)	143.69 (24.29)	144.91 (23.25)	< 0.001
DBP (mmHg)	77.50 (11.91)	76.41 (11.81)	75.12 (13.35)	77.05 (10.26)	0.001
Prevalent DM, *N* (%)	1127 (11.7%)	183 (23.0%)	33 (18.9%)	9 (17.3%)	< 0.001
Total chol (mmol/L)	6.15 (1.24)	6.37 (1.23)	6.37 (1.17)	6.14 (1.10)	< 0.001
HDL-chol (mmol/L)	1.38 (0.40)	1.33 (0.36)	1.37 (0.35)	1.33 (0.39)	< 0.001
Use of cardiac medication, *N* (%)	822 (6.5%)	128 (11.3%)	26 (12.5%)	4 (7.0%)	< 0.001
Use of lipid-lowering medication, *N* (%)	1249 (9.9%)	88 (7.7%)	14 (6.7%)	2 (3.5%)	0.061
Current smoking, *N* (%)	3083 (24.9%)	251 (22.3%)	33 (16.0%)	11 (19.6%)	0.006

Data as *N* (%) or mean ± SD. *P* value based on *χ*^2^ for categorical data, or one-way ANOVA for continuous data. *P* for trend

*AF* atrial fibrillation, *BMI* body mass index, *WC* waist circumference, *HC* hip circumference, *WHR* waist-to-hip ratio, *SBP* systolic blood pressure, *DBP* diastolic blood pressure, *DM* diabetes mellitus, *chol* cholesterol, *HDL* high-density lipoprotein

**Fig. 2:**
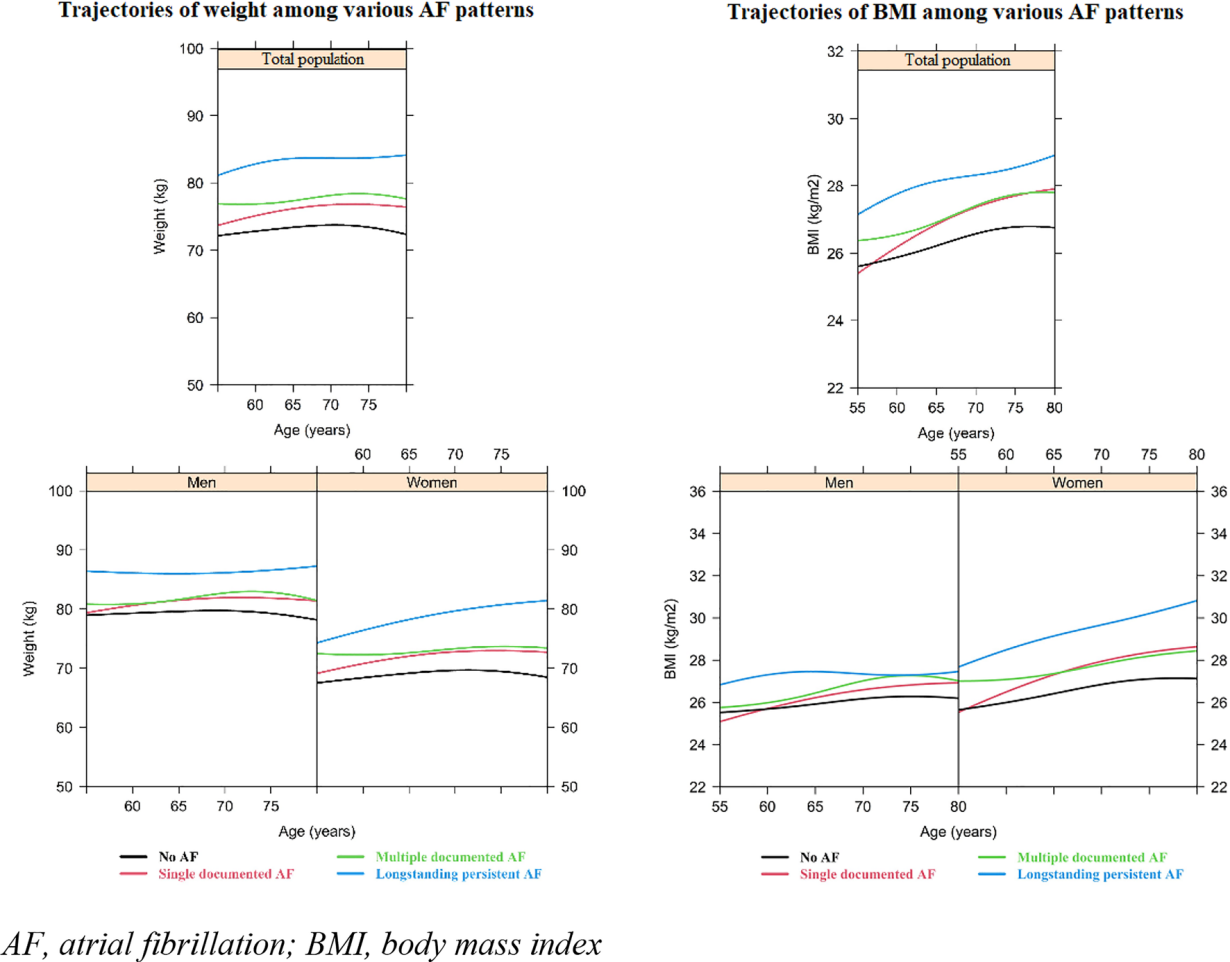
Evolution of weight and body mass index prior to various atrial fibrillation patterns in total population (upper bar) and among men and women (lower bar). *AF* atrial fibrillation, *BMI* body mass index

**Fig. 3:**
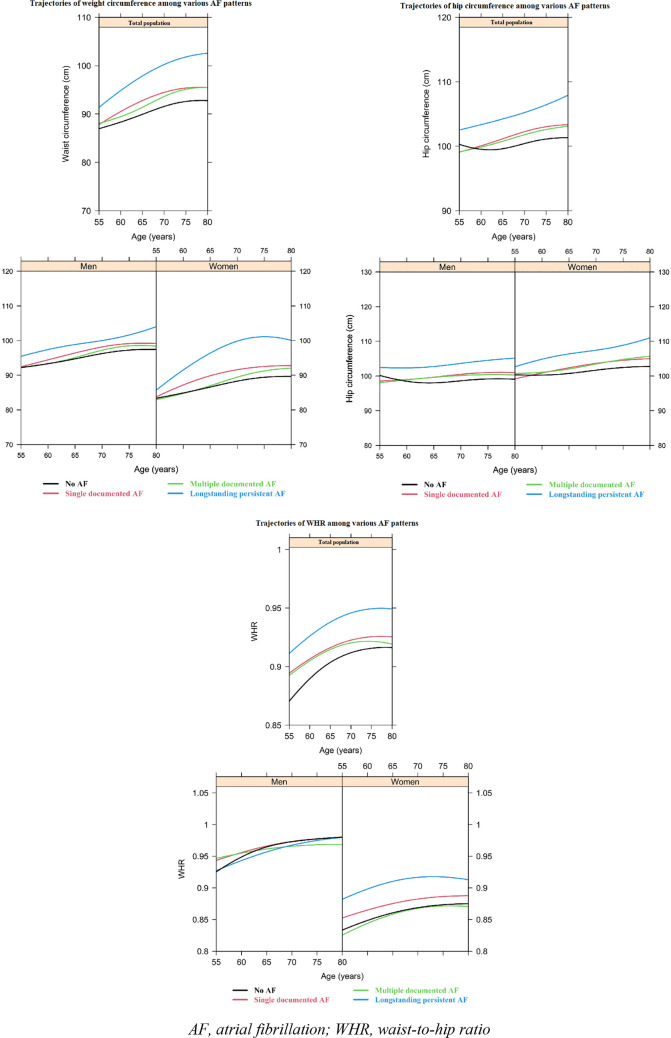
Evolution of anthropometric measures prior to various atrial fibrillation patterns in total population (upper bar) and among men and women (lower bar). *AF* atrial fibrillation, *WHR* waist-to-hip ratio

As depicted in Figs. 2 and 3, in the population ultimately diagnosed with long-standing persistent AF, the weight, BMI, waist circumference, hip circumference, and WHR were continuously higher than participants categorized with the other AF patterns. Participants in the long-standing persistent AF group also showed a similar SBP and DBP at a younger age, but these values increased more rapidly than in the other patterns. Looking at men and women separately, we found that men who later developed ‘long-standing persistent AF’ had a higher weight (around 5 kg) at all ages, as compared to the other men. For waist and hip circumference, there also appeared to be a difference of 3 cm between men categorized as ‘long-standing persistent AF’ and the other categories (Fig. 3).

**Fig. 4:**
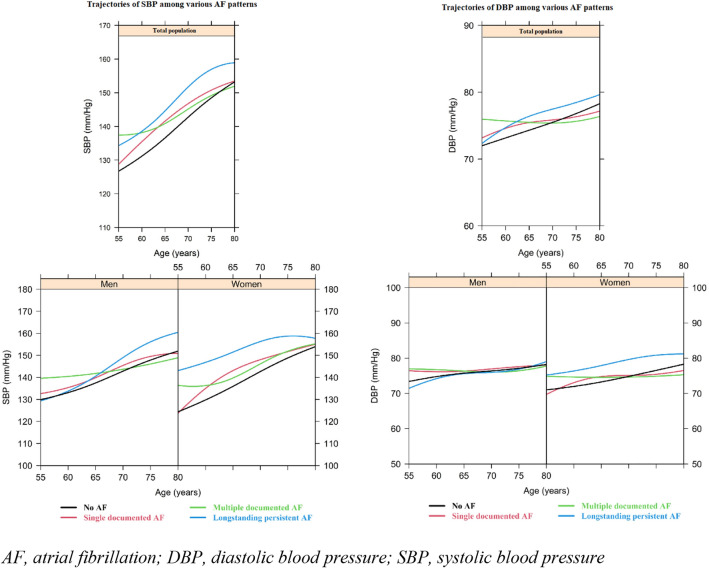
Evolution of systolic and diastolic blood pressure prior to various atrial fibrillation patterns in total population (upper bar) and among men and women (lower bar). *AF* atrial fibrillation, *DBP* diastolic blood pressure, *SBP* systolic blood pressure

In women, weight, BMI, waist circumference, and hip circumference all increased in a steeper manner in the ‘long-standing persistent AF’ category, as opposed to the other categories. Additionally, SBP levels were higher for the ‘long-standing persistent AF’ group as opposed to the other categories, but this difference attenuated when women grew older than 70 years (Fig. 4). Women categorized as’long-standing persistent’ or ‘multiple-documented AF episodes’ had higher DBP values than women categorized as ‘single-documented AF episode’ or women with no AF. However, DBP levels from the women in the ‘multiple-documented AF episodes’ group remained roughly the same, whereas DBP increased for women in all other categories, most noticeably in women who developed ‘long-standing persistent AF’, or did not develop AF. WHR was higher in women who developed ‘long-standing persistent AF’ at all ages, as opposed to all other categories.

## Discussion

We developed a classification for AF patterns in a general population. Overall, we found poorer trajectories for weight, BMI, weight circumference, hip circumference, WHR, and SBP in participants who developed ‘long-standing persistent AF’. In sex-stratified analyses, we found distinct poorer trajectories of weight, waist circumference, and hip circumference over time between men who developed ‘long-standing persistent AF’ and other AF patterns. In women, we additionally found worse cardio-metabolic risk profiles of BMI, SBP, DBP, and WHR between ‘long-standing persistent AF’ and the other patterns.

After a median follow-up of 9.4 years, 1402 participants (10%) developed at least one AF episode. Among participants who developed AF, 1137 (81.1%) were categorized as ‘single-documented AF episode’, 208 (14.8%) as ‘multiple-documented AF episodes’, and 57 (4.1%) as ‘long-standing persistent AF’.

Within the Framingham Heart Study, out of 478 participants with AF, 63 (10%) had no recurrence within two years, comparable to our ‘single documented AF episode’, 162 (26%) experienced a concurrent event within two years, comparable to our ‘multiple-documented AF episodes’, and 207 (34%) developed sustained AF, comparable to our ‘long-standing persistent AF’ [17]. In a similar method, the PREVEND study investigated predictors of AF recurrence within two years [18]. Out of 319 participants who developed AF, 103 (32%) had no recurrence, 158 (50%) had a self-terminating AF, and 58 (18%) had non-self-terminating AF. Most studies investigating progression of clinical AF patterns, however, find that around 50% of the AF cases develop a recurrent event, and that the majority of patients remain having short paroxysmal AF events [22–25]. Partly, our low number of recurrences can be explained by the large intervals between the consecutive research examinations. As the RS partly relies on hospital discharge letters and documentation by general practitioner, it is possible that asymptomatic AF episodes remain undiagnosed, and therefore are misclassified in our study. Moreover, if multiple AF episodes occur during hospitalization, this is often reported as one AF episode. However, this method of data collection and classification is representative of a real-world situation. Therefore, with this classification we set the grounds for large observational longitudinal cohort studies to investigate differences in etiology, pathophysiology, underlying risk factors, and prognosis between AF patterns in the general population.

We found distinct patterns for the evolution of various risk factors in the ‘long-standing persistent AF’ category, as compared to other AF patterns. Previous studies have tried to identify risk factors for AF progression and recurrence after cardioversion or ablation therapy [11, 23, 26–28]. However, these studies used the clinical classification, and are not performed in a general population. Additionally, to our knowledge we are the first study to investigate the longitudinal evolution of cardiovascular risk factors prior to AF patterns in a general population. A previous meta-analysis has shown that a higher BMI at baseline is significantly associated with a recurrent AF episode after ablation therapy [29]. Additionally, clinical evidence of baseline associations for cardiovascular risk factors, such as weight, BMI, and blood pressure, for patients with AF progression is inconclusive [11, 30]. It is thought that exposure to risk factors causes progressive atrial remodeling, eventually causing recurrent AF events, and progression to persistent and permanent AF [11, 31]. However, these studies investigated baseline levels, and evidence on the impact of different evolutions of these factors are lacking.

Obesity has previously been linked to atrial remodeling [32, 33]. Our findings support this, as participants categorized as ‘long-standing persistent AF’ had higher weight and BMI values at all ages preceding AF, especially in women. This could imply that longer exposure to obesity progressively impairs the cardiac function, eventually increasing AF recurrence risk and disease burden. Waist circumference, hip circumference, and WHR are indicators of body fat distribution. Higher waist circumference and WHR indicate central obesity, which has previously been associated with cardiovascular disease [33, 34]. Our findings imply that for men, the distribution of fat does not contribute to AF recurrences. However, in women, the waist circumference and WHR are continuously higher over all ages in participants categorized as ‘long-standing persistent AF’, as compared to the other patterns. Continuous exposure to central obesity may therefore be a larger risk in women than in men. A recent study showed that longer lasting elevated SBP and DBP were associated with an increased risk of AF, most noticeably in women [35]. Longer exposure to high blood pressures may cause cardiac dilatation, structural and electrical impairment, and eventually AF. This mechanism is further supported by our findings, as we found that women categorized as ‘long-standing persistent AF’ had higher SBP and DBP levels over all ages. In men, however, we found no differences between the various AF patterns.

While the exact pathophysiology of AF development is not clear, recent evidence suggests sex differences between underlying atrial remodeling mechanisms [36–38]. Sex hormones, such as estrogen and progestin, are suggested to reduce atrial remodeling, at least partly explaining the higher incidence of AF in men [38]. Additionally, atrial fibrosis could play a larger role in AF development in women than in men [19, 39]. In this light, the different mechanisms underlying AF between men and women can reflect differing associations with various risk factors. This is in line with our findings, as women in the ‘long-standing persistent AF’ group had consistently higher weight, BMI, blood pressures, and waist and hip circumferences as they aged, whereas in men, only differences in weight and waist and hip circumference were found. A possible explanation for this is the differences in etiology of hypertension between men and women. Hypertension in women is often related to sex hormones, such as estrogen and progestin [40, 41]. It is possible that these underlying differences in pathogenesis, and the generally steeper increase in blood pressure in women, carry additional risks for AF development and progression. Furthermore, a lack of awareness of the risks and prevalence of hypertension may still have a role in the treatment and surveillance of women at risk of AF [40].

Our findings are in line with the recent evidence suggesting prolonged exposure to risk factors is associated with AF development. With increased knowledge on risk factors of AF progression and recurrence, we can apply targeted therapy to improve risk profiles of individuals at risk of AF at an earlier stage [32]. Our standardized and simple categorization can be applied at population level, opening the door to large studies investigating AF patterns. Moreover, our findings accentuate the differences in AF etiology and underlying mechanisms across various AF patterns, and between men and women. This further underlines the importance of a sex-specific approach in AF prevention and management.

This study was embedded within the large population-based RS. Strengths of the RS include its prospective design, large study population and long follow-up period. Through extensive interviews by trained interviewers, periodical research center visits, and a continuous linkage with general practitioners and hospitals, AF events were carefully adjudicated. Additionally, the periodical visits to the research center allow for the availability of repeated measurements of risk factors over time. Therefore, longitudinal changes could be assessed. However, the large time intervals of 4–5 years between research visits, the lack of Holter monitoring, and the dependence on the accuracy and completeness of hospital and general practitioners’ databases could have led to some degree of misclassification. Moreover, while we actively encourage participants to remain in the Rotterdam study after a newly found diagnosis or disease period, participants may be inclined to opt out of the study after the development of a first AF episode. Therefore, the ‘single-documented AF episode’ pattern may contain participants who would otherwise be categorized as ‘multiple-documented AF episodes’, or ‘long-standing persistent AF’. Additionally, older participants or those in worse health were more likely to be classified as ‘single-documented AF episode’, as participants may have died within the 4–5 years between study visits. Lastly, the Rotterdam study mostly consists of participants from Caucasian descent and of older age. Our findings may therefore not be directly generalizable to other ethnicities or a younger population.

## Conclusion

We developed a standardized method to classify different AF patterns in the general population. Various AF patterns were accompanied by different trajectories of cardiovascular risk factors prior to AF diagnosis. Our findings further highlight sex differences in AF pathophysiology, and give insight into possible risk factors of various AF patterns.

## Data Availability

Data can be obtained upon request. Requests should be directed toward the management team of the Rotterdam study (datamanagement.ergo@erasmusmc.nl), which has a protocol for approving data requests. Because of restrictions based on privacy regulations and informed consent of the participants, data cannot be made freely available in a public repository.
